# Serpins in rice: protein sequence analysis, phylogeny and gene expression during development

**DOI:** 10.1186/1471-2164-13-449

**Published:** 2012-09-04

**Authors:** Sheila E Francis, Renan A Ersoy, Brian J Atwell, Thomas H Roberts

**Affiliations:** 1 Department of Chemistry and Biomolecular Sciences, Macquarie University, North Ryde, NSW, 2109, Australia; 2 Department of Biological Sciences, Macquarie University, North Ryde, NSW, 2109, Australia; 3 Department of Plant and Food Sciences, Faculty of Agriculture and Environment, University of Sydney, Sydney, NSW, 2006, Australia; 4 Green Bio Research Center, Korea Research Institute of Bioscience and Biotechnology (KRIBB), 111 Gwahangno, Yuseong-gu, Daejeon, 305-806, Korea

**Keywords:** Serpin, Protease inhibitor, Rice, *Oryza sativa*, *Arabidopsis thaliana*, Expression

## Abstract

**Background:**

Most members of the serpin family of proteins are potent, irreversible inhibitors of specific serine or cysteine proteinases. Inhibitory serpins are distinguished from members of other families of proteinase inhibitors by their metastable structure and unique suicide-substrate mechanism. Animal serpins exert control over a remarkable diversity of physiological processes including blood coagulation, fibrinolysis, innate immunity and aspects of development. Relatively little is known about the complement of serpin genes in plant genomes and the biological functions of plant serpins.

**Results:**

A structurally refined amino-acid sequence alignment of the 14 full-length serpins encoded in the genome of the japonica rice *Oryza sativa* cv. Nipponbare (a monocot) showed a diversity of reactive-centre sequences (which largely determine inhibitory specificity) and a low degree of identity with those of serpins in *Arabidopsis* (a eudicot). A new convenient and functionally informative nomenclature for plant serpins in which the reactive-centre sequence is incorporated into the serpin name was developed and applied to the rice serpins. A phylogenetic analysis of the rice serpins provided evidence for two main clades and a number of relatively recent gene duplications. Transcriptional analysis showed vastly different levels of basal expression among eight selected rice serpin genes in callus tissue, during seedling development, among vegetative tissues of mature plants and throughout seed development. The gene *OsSRP-LRS* (Os03g41419), encoding a putative orthologue of *Arabidopsis* AtSerpin1 (At1g47710), was expressed ubiquitously and at high levels. The second most highly expressed serpin gene was *OsSRP-PLP* (Os11g11500), encoding a non-inhibitory serpin with a surprisingly well-conserved reactive-centre loop (RCL) sequence among putative orthologues in other grass species.

**Conclusions:**

The diversity of reactive-centre sequences among the putatively inhibitory serpins of rice point to a range of target proteases with different proteolytic specificities. Large differences in basal expression levels of the eight selected rice serpin genes during development further suggest a range of functions in regulation and in plant defence for the corresponding proteins.

## Background

Serpins constitute one of more than 80 families of protease inhibitors in nature [1] but are the dominant family in animals [2]. Serpins have been shown to be involved in a remarkable diversity of physiological processes in humans and in distinct model animal systems [3]. Most animal serpins act biochemically as irreversible inhibitors of specific endogenous serine (less commonly cysteine) proteinases [2]. For example, the well-studied mammalian serpin, antithrombin (SERPINC1), is an inhibitor of several of the activated forms of blood coagulation factors including thrombin (Factor IIa) as well as other serine proteinases of the chymotrypsin family [4].

The nomenclature of the secondary structural elements of serpins (using human α_1_-antitrypsin as a model) was defined more than 20 years ago [5]. A typical serpin molecule is characterised by three β-sheets (A–C), eight to nine α-helices (A–H) and a reactive-centre loop (RCL). The RCL displays an extended, exposed proteinase bait protruding from the body of the serpin scaffold and is one of several features critical for the function of inhibitory serpins [6]. The first X-ray crystal structure of a plant serpin—that of *Arabidopsis thaliana* AtSerpin1 (locus At1g47710) in the native, stressed conformation—was obtained recently and shown to be consistent with the description above but to also display plant-specific features [7].

Serpins inactivate their target proteinases using a unique mechanism involving large conformational change and a loss of structural metastability of the serpin to form a kinetically stable, covalent complex with the target enzyme [8]. In the native, stressed conformation of the serpin, the RCL presents a bait sequence to the proteinase [9]. (Note: RCL residues *N*-terminal from the protease cleavage site are defined as P1, P2, P3, etc., while those on the *C*-terminal side are termed P1′, P2′, P3′, etc. [10]). Upon cleavage of the RCL at the P1-P1′ bond of the reactive centre by the proteinase, the RCL inserts as an extra strand into the main β-sheet of the serpin molecule and the covalently attached proteinase [11] is flung to the opposite end of the serpin [12,13]. The conformational change in the serpin from native to cleaved is known as the stressed-to-relaxed (S→R) transition [14]. The proteinase is crushed against the body of the serpin, thereby distorting the active site of the enzyme and preventing hydrolysis of the peptide bond between the active-site Ser (or Cys) of the proteinase and the P1 residue of the serpin [13,15].

The inhibitory specificity of a serpin depends largely on the identity of residues in the reactive centre, particularly P1, although additional residues from P6 to P3′ [16,17] as well as exosite interactions [8] may influence the efficiency of proteinase inhibition. A minority of serpins have lost their inhibitory activity over the course of evolution [18] and have adapted to other roles. For example, the mammalian non-inhibitory serpins corticosteroid binding globulin (CBG; SERPINA6) and thyroxin-binding globulin (TBG; SERPINA7) bind steroid hormones in the blood and release these compounds at specific sites via a mechanism involving cleavage of the RCL [19,20].

While dozens of intra- and extracellular animal serpins have been functionally characterised, relatively little is known about the functions of serpins in plants [21-23]—nor indeed in unicellular eukaryotes [24-26] and prokaryotes [25,27-29]. Within the Viridiplantae, serpin genes have been identified in unicellular green algae, bryophytes, gymnosperms and flowering plants [22]. Testing via *in vitro* inhibition assays has shown that nearly all plant serpins studied are potent inhibitors of specific mammalian serine proteinases [30-36]. AtSerpin1 from *Arabidopsis* has been shown to inhibit Metacaspase 9 (AtMC9), an endogenous cysteine proteinase, *in vitro*[37]. More recently, the major *in vivo* target proteinase for AtSerpin1 was identified as the papain-like cysteine proteinase RESPONSIVE TO DESICCATION-21 (RD21) [7], an enzyme that also has transpeptidase activity [38]. Two other *Arabidopsis* serpins, AtSRP2 (ArathZ2; At2g14540) and AtSRP3 (ArathZ1; At1g64030), are associated with plant responses to alkylating DNA damage [39]. Serpins found at high concentrations in seeds (up to 4% total protein in wheat grain [34]) are assumed to provide direct defence against exogenous proteinases from insects and other organisms that attack the endosperm and other seed tissues [21,22,34].

The fully sequenced genome of japonica rice (*Oryza sativa* cv. Nipponbare) has been analysed to identify all serpin genes using PSI-BLAST searching [21,22]. A total of 14 genes encoding full-length serpins (340–440 amino-acid residues) were identified, eight of which were associated with evidence for expression based on publicly available ESTs, microarrays and proteomics data. The RCL sequences of these serpins were aligned to determine whether each sequence was likely to represent an inhibitory or a non-inhibitory serpin [22]. One of the rice serpins, here named OsSRP-PLP (Os11g11500), featured an RCL sequence that strongly suggested it was a non-inhibitory serpin, while two of the other serpins were less confidently predicted as being non-inhibitory. For the eleven putatively inhibitory serpins, the reactive-centre P2-P1′ sequence was different in each case, with considerable diversity associated with the critical P1 residue. Indeed, positively charged (Arg and Lys), small uncharged (Ala, Gly, Ser) and hydrophobic (Leu, Met) residues were identified at this position [22].

The aims of this study were to create a new nomenclature for the rice serpins, which could be extended to other species, and to describe the complement of rice serpin proteins, examine their phylogeny and measure the basal expression levels of their genes during plant development. We: (i) produced a phylogenetic analysis of the 14 full-length serpins in *O. sativa* cv. Nipponbare based on a carefully curated alignment of protein sequences (derived from revised gene models for several of the serpins); (ii) showed the extent to which genomic PCR using primer sets designed for *O. sativa* cv. Nipponbare could amplify serpin genes in other varieties of *O. sativa* and in wild species of *Oryza*; (iii) determined the basal expression pattern of eight selected rice serpin genes in callus, seedlings during development, organs of mature plants and in developing seeds, and (iv) compared the rice serpins to those in *Arabidopsis* to identify putative orthologues.

## Methods

### Amino-acid sequence alignment and phylogenetic analysis

Sequences were aligned using ClustalX [40] and edited by hand to ensure alignment of the residues as constrained by the length of the RCL [41]. The alignment was augmented with the amino-acid sequence of AtSerpin1 along with corresponding secondary structure assignments based on the X-ray crystal structure of the native conformation [7]. For construction of the phylogenetic tree, an msf file was generated and imported into PAUP v4.0b10 [42]. All sites in the alignment with gaps in any sequence were excluded and the remaining sites processed using parsimony (default settings in PAUP). A tree was constructed with 1000 bootstrap trials. The resulting .phb file was imported into TreeView X v0.5.0 [43] and a rectangular cladogram constructed.

### Plant growth conditions

#### Origin and sterilisation of rice seeds and growth of seedlings on solid media

*Oryza sativa* cv. Nipponbare seeds were obtained from Dr. Alexander Johnson, University of Adelaide, Australia. Other *O. sativa* varieties were obtained from SunRice (Leeton, NSW). The *O. australiensis* seeds were obtained via the Australian Plant Genetic Resource Information Service (ATCGRC #122; http://www2.dpi.qld.gov.au/extra/asp/AusPGRIS/) and the *O. meridionalis* seeds were collected from Cape York, Queensland (15° 41^′^ S and 145° 2^′^ E).

Seeds were dehulled and washed with 0.5 mM CaCl_2_ for 30 min on a shaker at low speed. The CaCl_2_ solution was discarded and the seeds were incubated in 70% ethanol for 30 s. After three washes with sterile distilled water, the seeds were placed in 10% commercial bleach for 3 min followed by a single wash in water and incubation in HgCl_2_ (1000 ppm) solution for 3 min. Finally the seeds were rinsed in sterile distilled water (5 × 1 min).

Sterile cylindrical Perspex jars (15.5 cm high × 6.8 cm diameter) containing sterile medium were prepared by adding ~50 ml 1× Murashige and Skoog (MS) salt medium and 0.8% agar into each jar, placing non-absorbent cotton wool in the air vent in the top of each jar and autoclaving the jars at 121 psi for 20 min. The sterilised seeds were placed on top of the media in the jars (in a laminar flow cabinet), which were then kept in the dark for 5 d. Since the seeds germinated ~3 d after imbibition, seedlings collected immediately after the 5-d imbibition are referred to as 2-d-old seedlings. The remaining of the seedlings were transferred into sterile jars containing MS medium. The jars were placed in a growth chamber (Thermoline) with a cycle of 16 h at 28°C in the light and 8 h at 15°C in the dark. The light intensity was 215 μmoles m^-2^ s^-1^ provided by GE Polylux XL fluorescent tubes (model F38U/840 CVG).

#### Growth of plants in soil and isolation of mature plant tissues and developing seeds

Rice seeds were dehulled, soaked in 0.5 mM CaCl_2_ for 30 min and sown in soil (equal parts of a fine-textured krasnozem from Robertson, NSW, a silty clay-loam from Bungendore, near Canberra, ACT, and general potting mix (Australian Native Landscapes)) in pots. The pots were placed in trays of water in a temperature-controlled glasshouse, 28°C for ~16 h (day) and 15°C for ~8 h (night). Plants were grown for 6–8 weeks from germination. Samples of leaf, stem, root and root tip (cut ~2 mm from the end of the roots) were collected, frozen in liquid nitrogen and stored at −80°C. Developing seeds were collected at 5, 10, 15, 20, 30 and 40 d post-anthesis, frozen in liquid nitrogen and stored at −80°C.

### Isolation of DNA and genomic PCR

Seeds from the *O. sativa* cultivars and wild Australian relatives were sown in soil as described above. After 6–8 weeks, leaves from each variety were harvested, frozen in liquid nitrogen and ground using a pre-chilled mortar and pestle. Total genomic DNA was extracted using the DNeasy® Plant Mini kit (Qiagen) according to the manufacturer’s instructions. The PCR cycle and primers used were the same as described for the semi-quantitative RT-PCR.

### Isolation of RNA and semi-quantitative RT-PCR

Total RNA was extracted from 100 mg plant tissue using an RNeasy Plant Mini kit (Qiagen) according to the manufacturer’s instructions. For RT-PCR, total RNA (10 μg) was treated with DNA-free™ (1 unit of RNase-free DNase; Ambion). The first-strand cDNAs were synthesised using 5 μg DNase-treated total RNA with oligo(dT) (50 μM), 200 U Superscript III reverse transcriptase (Invitrogen), 10 mM dithiothreitol (DTT), 500 μM of each dNTP and 20 U RNase inhibitor. For PCR amplification, the following components were combined in a 0.2-ml tube: cDNA template, 1× reaction buffer (Thermo Scientific), 2.0 mM MgCl_2_ (Thermo Scientific), 0.2 mM dNTPs (Qiagen), 0.8 M betaine (Sigma), 0.8 U Taq polymerase (Red Hot Taq from Integrated Sciences) and 10 pmol of each primer (forward and reverse primers as listed in Table 1). Sterile Milli-Q water was added to give a final volume of 20 μl. The PCR cycle was 94°C for 5 min, 35 cycles of 94°C for 30 s, 55°C for 30 s and 72°C for 2 min and a final extension step of 72°C for 5 min with a Px2 thermal cycler (Thermo Electron Corporation).

**Table 1:** Primers used for semi-quantitative RT-PCR

**Primer**	**Sequence**	**Amplicon size (bp)**
*OsSRP-QKG* fwd	5^′^-TGCCCCGAGCCGCATTCTAC-3^′^	359
rvs	5^′^-TTGCATCATAACCACGGCGG-3^′^	
*OsSRP-LGC* fwd	5^′^-GACACCGGCCGCCTCTTCTC-3^′^	383
rvs	5^′^-GCGCAGCCAAGGGTCATGAC-3^′^	
*OsSRP-LRS* fwd	5^′^-AAGCTTCCATACCAGCAAGG-3^′^	395
rvs	5^′^-GGAGCTGACCTAAGTGTGATC-3^′^	
*OsSRP-FRS* fwd	5^′^-GCTTATGGCTTGACCACAAG-3^′^	451
rvs	5^′^-CACTGCCTTCCATCACGTAG-3^′^	
*OsSRP-PTY* fwd	5^′^-ACGACGGCCAGGTCCACTTC-3^′^	398
rvs	5^′^-GCTATAGGTCGGGCTGCAAC-3^′^	
*OsSRP-PLP* fwd	5^′^-AGCAAGGAAAGAATGAAAGG-3^′^	362
rvs	5^′^-CAAGCCCATTGATACTGAAG-3^′^	
*OsSRP-FAS* fwd	5^′^-GCTTGTGTGAGCGCGAGGAC-3^′^	399
rvs	5^′^-GAAGGTAAGACAAACCGCGG-3^′^	
*OsSRP-FLC* fwd	5^′^-GATGGTGGTGACATCACTCC-3^′^	423
rvs	5^′^-CAAGAACTTCATCGTGCAGG-3^′^	
*Actin* fwd	5^′^-TCCATCTTGGCATCTCTCAG-3^′^	418
rvs	5^′^-GTACCCGCATCAGGCATCTG-3^′^	

BLASTN searches against the rice genome using the primers as query sequences were performed to check for the possibility of non-specific hybridisation. fwd = forward; rvs = reverse.

Primers were obtained from Sigma. Primer pairs and their expected amplicon sizes for semi-quantitative RT-PCR and genomic PCR experiments are shown in Table 1. The regions chosen for the forward and reverse primers corresponded to ~400 bp upstream from the DNA sequences encoding the hypervariable RCL region and to the hypervariable RCL region itself, respectively. The primer pairs were first tested using genomic PCR. Single PCR products of the expected sizes were obtained for each of the eight primer pairs and no bands were present in any of the minus-template controls (results not shown). Amplicon sequencing using a 3130X/Genetic Analyzer (Applied Biosystems) followed by BLASTN searching against the “Genes in MSU Osa1 Rice Pseudomolecules – Genomic” database using default parameters (including Expect threshold = 10) at the MSU Rice Genome Annotation website (http://rice.plantbiology.msu.edu/) confirmed that all of the PCR products amplified corresponded to the expected genes (not to other serpin genes, serpin pseudogenes or unrelated genes; results not shown).

### Real-time RT-PCR

For quantitative real-time RT-PCR, the QuantiTect SYBR® Green PCR kit (Qiagen) was used with a LightCycler® (Roche). As performed above for the semi-quantitative RT-PCR primers, real-time primers (Table 2) were tested by genomic PCR followed by BLASTN searching using the sequencing products as queries. All of the PCR products amplified corresponded to the expected genes (results not shown).

**Table 2:** Primers used for real-time RT-PCR

**Primer**	**Sequence**	**Amplicon size (bp)**
*OsSRP-QKG* fwd	5^′^-ACATGCGGAAGCTGGGCGTGA-3^′^	167
rvs	5^′^-TTGCATCATAACCACGGCGGTG-3^′^	
*OsSRP-LGC* fwd	5^′^-ACAAGACGAACGCGGCGGAGAC-3^′^	133
rvs	5^′^-TGGTAGACGGCCGACACGACGA-3^′^	
*OsSRP-LRS* fwd	5^′^-ACGCGGCAAGTTACTGTCGGGC-3^′^	228
rvs	5^′^-TGCAGCAGCAGCCTCAGTCCC-3^′^	
*OsSRP-FRS* fwd	5^′^-GGCTGCGAAGCTGAACTCTGAAC-3^′^	197
rvs	5^′^-TCTCTGGAGAACCCACCATCCCA-3^′^	
*OsSRP-PTY* fwd	5^′^-AGCGAGCAGGAAGTCTCCCCG-3^′^	155
rvs	5^′^-GGCGGCGTGTTCACACTCACA-3^′^	
*OsSRP-PLP* fwd	5^′^-CCTCCGGGAAGCTGAATTCTCTG-3^′^	220
rvs	5^′^-TACTGTTCCAGAGACCTCCTCCC-3^′^	
*OsSRP-FAS* fwd	5^′^-TCAAGCCGTTCGTGGCGGACC-3^′^	170
rvs	5^′^-CGCCGCCGATGCGAAGGTAAG-3^′^	
*OsSRP-FLC* fwd	5^′^-AAGATGGCAGTGGCGTCGTCCG-3^′^	303
rvs	5^′^-CTTCATCGTGCAGGCCGTGG-3^′^	

BLASTN searches against the rice genome using the primers as query sequences were performed to check for the possibility of non-specific hybridisation. fwd = forward; rvs = reverse.fwd = forward; rvs = reverse.

The samples were diluted to 50–125 ng μl^-1^ and run as three technical replicates (triplicates). The CP (crossing-point) values were detected by the LightCycler® Software v.4.0. Expression profiles were compared with that of the housekeeping gene, *Actin* (Os03g50885). In some experiments a second housekeeping gene, *GAPDH* (Os04g40950) was also included.

To allow presentation of real-time RT-PCR data for poorly-expressed genes (e.g. *OsSRP-PTY*) and highly-expressed genes (e.g. *OsSRP-LRS*) on single figures, the CP values from the LightCycler® analysis were firstly transformed by assigning (arbitrarily) a CP value of 20.00 to a relative transcript abundance of 100,000 units (expression value = 100,000 × 2^(20-x)^, where x is the CP value obtained for the gene analyzed) and then plotted on a log_10_ scale on the Y-axis. The CP value of 20.00 was convenient because the basal expression of *Actin* (the most highly expressed gene measured) corresponded to CP ~20. To assist in interpreting the relative transcript abundance values plotted on the log_10_ scale, the values in each figure are also given in an integrated table immediately below each plot.

## Results

### A new rice serpin nomenclature

In a detailed review of plant serpins conducted previously, each of the 14 full-length serpins encoded in the *O. sativa* cv. Nipponbare genome was assigned a unique name, such as OrysaZ2a [22]. Since serpins from ~60 plant species were compared in the review, serpin names included a five-letter abbreviation for the Latin name (e.g. Orysa for *Oryzasativa*). The “Z” designation was derived from “Protein Z”, the name given to barley grain serpins [44] before the word “serpin” was coined [45]. Numbers in the names were based on a combination of degree of overall identity and of RCL length and sequence to previously named plant serpins. Where no match to a previously named serpin was found, the number of that serpin was avoided (thus, for example, none of the rice serpins was named OrysaZ7 because none had sufficient general similarity as described above to barley serpin Z7). Recently, one of the rice serpins was named OsSerpin [46] but a more appropriate name may have been OsSerpin1 because this particular serpin is the reactive-centre match and putative orthologue of AtSerpin1, as named earlier [37].

Here we created a new, alternative nomenclature for rice serpins by (i) shortening the first part of the name to “Os” for *Oryza sativa*, (ii) removing the “Z” designation, (iii) adding “SRP” for “serpin”—nomenclature consistent with recent naming of *Arabidopsis* serpins [39]—and (iv) adding the one-letter codes for the amino-acid residues corresponding to the canonical P2-P1′ sequence of the RCL in each case. The assignment of these residues was determined by counting residues *C*-terminal to the highly conserved Glu at P17. For example, the rice serpin encoded at the locus Os03g41419 was given the name OsSRP-LRS (Additional file 1: Table S1). Corresponding gene names are given in italics. The reactive-centre sequence was adopted in preference to the numbering system used previously [22] because the former contains functional information lacking in the latter. The inclusion of “SRP” in the new alternative names will also assist readers in identifying proteins as serpins.

### Revised rice-serpin gene models

**Figure 1:**
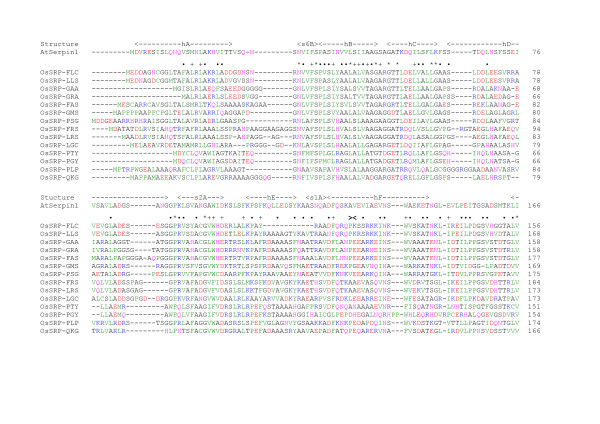
**Amino acid sequence alignment for the 14 full-length serpins in rice (*Oryza sativa *cv. Nipponbare) with the *Arabidopsis *AtSerpin1 sequence and secondary structure.** The *Arabidopsis* AtSerpin1 sequence and secondary structure assignments were from the X-ray crystal structure [PDB: 3LE2] [7]. Rice sequences were obtained from the Rice Genome Annotation Project (Michigan State University), edited where required according to the text and aligned using ClustalW (accurate) using default parameters (gap opening penalty = 10, gap extension = 0.05, Blosum30 series protein weight matrix). Note the sequence shown for OsSRP-PLP does not include the 45-residue *N*-terminal extension supported by full-length cDNA evidence as described in the text. Residues are coloured according to physico-chemical properties: Black, small; Green, medium-sized and large hydrophobic; Pink, polar; Red, negatively charged; Blue, positively charged [47]. *, identical residues in all rice serpins; +, conserved residues in all rice serpins (according to colour scheme above); ·, conserved residues in 11–13 of the 14 rice serpins (according to colour scheme above); **><**, intron-exon boundary for all rice serpins in alignment except OsSRP-QKG, which lacks an intron.

We attempted to revise sequence models when they did not fit the transcript-based evidence. We also assumed that if no transcript evidence was available, the gene model should be consistent with the gene models for the other serpins in the same species. Based on the analysis conducted here, the putative products encoded by serpin genes *OsSRP-LGC* (Os01g56010), *-FRS* (Os03g41438), *-PTY* (Os04g45110), *-PGY* (Os04g45120), *-FAS* (Os11g12460), *-GMS* (Os11g12520), *-LLS* (Os11g13530) and *-FLC* (Os11g13540) had been annotated with correct protein lengths in the Rice Genome Annotation Project database (http://rice.plantbiology.msu.edu/). OsSRP-QKG (Os01g16200) had a predicted protein length of 423 aa in the database, suggesting that this serpin would have a *N*-terminal extension relative to most of the other serpins. To our knowledge no transcript evidence was available to support this extension. Since an alternative START codon was available to give a protein with a length compatible with almost all the other rice serpins, 25 residues were removed from the *N*-terminus of OsSRP-QKG for the sequence alignment so that the protein sequence begins with MAPP rather than MAAL (Figure 1). The serpin OsSRP-LRS (Os03g41419) was incorrectly annotated in the database as producing a protein of 719 aa in length. The serpin sequence was edited by retaining the 137 residues at the N-terminus from MADD to FQTK but removing 72 residues (corresponding to two erroneous exons) starting from WLLL and ending in TSGK. The following 252 residues beginning at AAEV and ending at VGHV were retained. Finally, the last seven residues which, according to the erroneous gene model, are AAEVLGQ, were changed to VNPLLAA in accordance with translation of an available cDNA (accession no. AK243629). Thus the total length of OsSRP-LRS is 137 + 252 + 7 = 396 residues, consistent with the protein length predicted elsewhere based on translation of ESTs and cDNAs [22]. The Rice Genome Annotation Project database indicated that *OsSRP-LRS* (Os03g41419) contained four introns but the corrected sequence contains only one intron, consistent with the other rice serpins (Figure 1). The fifth exon in the erroneous gene model is associated with a separate cDNA sequence (accession no. AK121227). When translated, this cDNA gives a sequence beginning with LYFK and ending with LLAV, with a total length of 218 aa. This represents a partial second serpin sequence and includes an RCL distinct from that encoded by exon 4 in the original gene model.

OsSRP-PLP (Os11g11500) had a predicted protein length of 439 aa in the database. The *N*-terminal extension (relative to the other rice serpins) is supported by a full-length cDNA sequence (AK287588) and several ESTs (including CI370534 and CI410938). This is the only rice serpin for which a full-length cDNA supports a substantial *N*-terminal extension. For the sequence alignment (Figure 1), the first 45 residues (MQVSSYLRRALRRPPFPAGDANHRRLSSAPAPKPEAPAEAMPPPP) were removed from the *N*-terminus so that the sequence began at MPTRPW and contained 394 aa residues, consistent with the protein length predicted earlier [22]. OsSRP-PSG (Os11g11760) was annotated in the database as producing a protein of 452 aa. This sequence was edited by removing the 30 aa residues that corresponded to an (invalid) translation of the intron—found at a conserved site [22]—in this gene and the five contiguous and non-conserved Ala residues (almost certainly an artefact) that were present later in the sequence. This gave the predicted protein a length of 417 aa (Figure 1). *OsSRP-PTY* (Os11g12410) and *-PGY* (Os11g12420) were annotated in the database as containing two introns and producing proteins of 510 aa and 524 aa, respectively. No full-length cDNAs support these models. The corrected protein lengths are 393 and 398 aa for OsSRP-PTY and -PGY, respectively, with both of the corresponding genes containing one intron. Relative to the database sequence, 117 aa residues were removed from the *N*-terminus of OsSRP-PTY and 126 aa residues from the *N*-terminus of OsSRP-PGY [22]. The current gene model for OsSRP-GMS encodes a serpin with a substantial *C*-terminal extension. There is no STOP codon (TAG, TAA or TGA) earlier in the sequence than the existing one (TAG, which ends the OsSRP-GMS sequence at FVGV; Figure 1).

### Amino-acid sequence alignment and phylogenetic analysis

Alignment of the edited amino-acid sequences showed several highly conserved regions (Figure 1), as was expected based on alignments of serpins from other organisms. These regions include the hinge of the RCL, the breach, shutter and other regions critical for generation of the serpin fold [48]. The majority of differences between the rice serpin sequences are the presence/absence of additional amino-acid residues between conserved regions. Disregarding the putative 45-aa *N*-terminal extension of OsSRP-PLP (as discussed above), the *N*-terminus of the serpins (Figure 1 – up to the Asn conserved in all 14 serpins in the middle of the first line) varies in length from 19 to 44 aa. Two of these serpins have three consecutive negatively charged residues, which conceivably could serve as a binding motif for interacting molecules. The *C*-terminus of OsSRP-GMS (Os11g12520) is substantially longer (by ~30 residues) than the *C*-termini of any of the other serpins and includes a sequence of eight negatively charged residues, which might also serve as a binding motif.

Features of the AtSerpin1 X-ray crystal structure include a relatively long loop joining β-strands s2B and s3B, which contains a plant-specific motif between Tyr-225 (the conserved breach tyrosine) and the hydrophobic core residue Phe-234 [7]—see Figure 1 for these residues. An alignment of 67 expressed plant serpin sequences showed that the motif Y*XX*G*X*D*X*R*X*F was present in 54 of these sequences, with an additional eight sequences containing conservative variations of the motif [7]. The conserved Asp-230 and Arg-232 of this motif in AtSerpin1 form a network of hydrogen bonds that links the s2B-s3B junction to the loop connecting helix hD and β-strand s2A. These interactions stabilise this loop region, which is otherwise disordered in many other serpin structures [7]. The breach Tyr is present in all the 14 rice serpins while the hydrophobic core Phe is found in eight of the 14 sequences, with another hydrophobic residue (Tyr or Leu) in the other six serpins (Figure 1). The only rice serpins in which the motif is conserved—including the Asp and Arg mentioned above—are OsSRP-FRS and -LRS (putative orthologues of AtSerpin1). The limited conservation of the plant-specific motif in rice serpins may be partly a reflection of the somewhat biased nature of the 67 serpin sequences mentioned above, since nearly half of these sequences were those of LR serpins. While the AtSerpin1 sequence between (and including) the conserved Asp-230 and Arg-232 is 10 amino acids long (see motif above), the length of the corresponding region in the rice serpins ranges from 10 amino acids (OsSRP-FRS and -LRS) to 28 amino acids in OsSRP-FLC and 36 amino acids in OsSRP-LLS. Additional X-ray crystal structures of plant serpins will be required to determine whether the variation in the length of this loop is structurally important.

**Figure 2:**
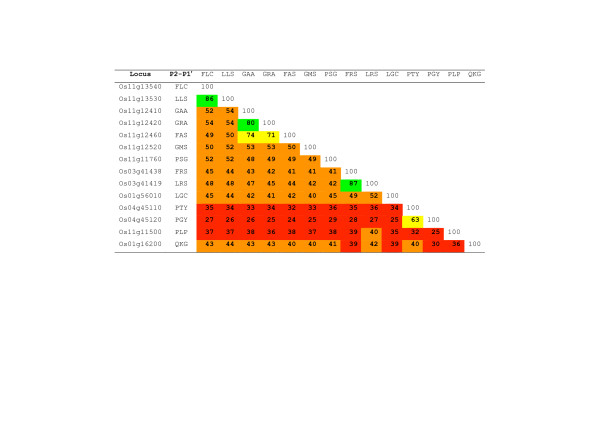
**Amino acid identity matrix for the 14 full-length serpins in rice.** An edited amino acid alignment (Figure 1) was entered into ClustalX v2.0 as an .aln file and the percent identities calculated by the program. Colours represent bands of percent identity: red, 20–39%; orange, 40–59%; yellow, 60–79%; green, 80–99%.

The degree of identity between the rice serpins ranged from 24% to 87%, with an average value for all pair-wise comparisons of 42.9% (Figure 2). The pairs of serpins with the highest levels of identity were OsSRP-LRS and -FRS (87%) and OsSRP-LLS and -FLC (86%). Members of these pairs have similar reactive centres and represent neighbouring genes (on chromosomes 3 and 11, respectively), suggesting that they arose through relatively recent gene duplications, as supported by the phylogenetic analysis (see below).

**Figure 3:**
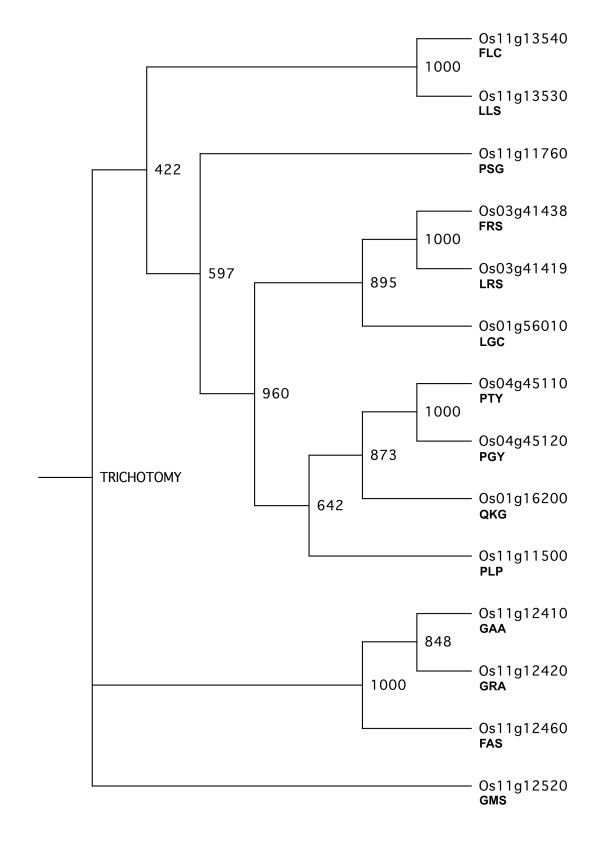
**Phylogenetic tree based on protein sequences of the 14 rice serpins.** A neighbour-joining tree using the 14 serpins in rice was constructed using the ClustalX v2.0 program and presented using TreeView X v0.5.0 [43]. Bootstrap values from 1000 bootstrap trials are given on the nodes. Locus numbers and P2–P1^′^ codes are used as labels for the serpins.

Phylogenetic analysis of the rice serpins based on amino-acid sequences resulted in a trichotomy at the tree base, with one of the serpins (OsSRP-GMS; Os11g12520) alone on one of the three main branches (Figure 3). The serpins on the top-most main branch of the tree were moderately well resolved into several smaller clades (boot-strap values 422 to 960) while those on the middle branch were very well resolved (boot-strap value 1000) (Figure 3).

### Detection of putative orthologues of *Oryza sativa* cv. Nipponbare serpin genes in other varieties of *Oryza sativa* and other species of *Oryza*

As the genome sequences of the other varieties and wild species are not known, a genomic PCR experiment was conducted to test whether primers designed to amplify a fragment of eight of the serpin genes in *O. sativa* cv. Nipponbare could be used to detect serpin genes in these rices. Only serpin genes with sequences at the sites of primer hybridisation identical or near-identical to those in Nipponbare were likely to be amplified by the primers.

**Figure 4:**
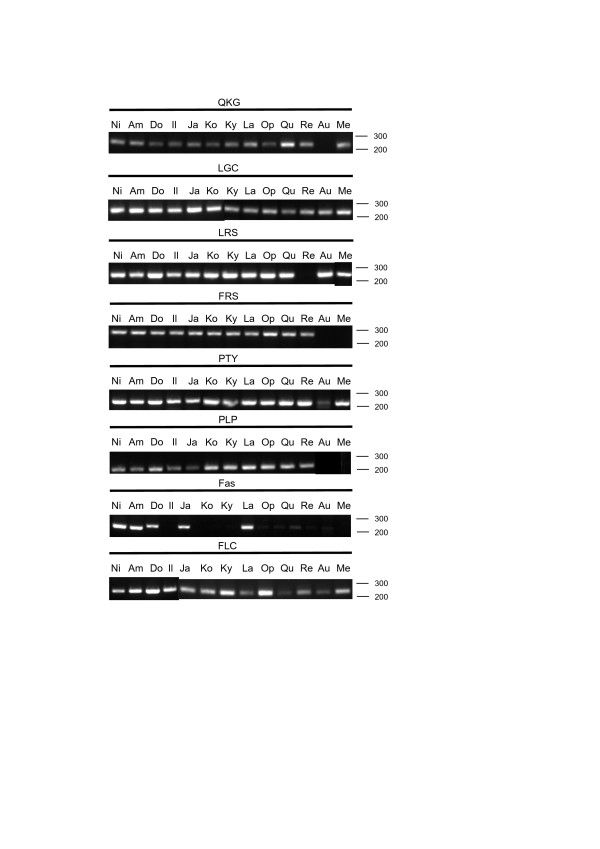
**Evidence for conservation of serpin genes in a variety of *O. sativa *cultivars and wild *Oryza *species.** Primer sets used to amplify eight serpin genes in Nipponbare (labelled according to P2–P1^′^ sequence) were used to amplify segments of putatively corresponding genes in a range of *O. sativa* varieties and wild rice species using genomic PCR. Ni = Nipponbare; Am = Amaroo; Do = Doongara; Il = Illabong; Ja = Jarrah; Ko = Koshihikari; Ky = Kyeema; La = Langi; Op = Opus; Qu = Quest; Re = Reiziq; Au = *O. australiensis*; and Me = *O. meridionalis.*

Amplicons were generated with primers for *OsSRP-LGC*, *-PTY*, *-PLP* and *-FLC* in all of the rices (Figure 4). *OsSRP-QKG* primers amplified a product of the expected size in all of the rices except *O. australiensis*, while *OsSRP-LRS* was not amplified in cv. Reiqiz. *OsSRP-FRS* was not detected in *O. australiensis* and *O. meridionalis*. *OsSRP-FAS* was amplified in cv. Nipponbare, Doongara and Kyeema. Limited amplification using the *OsSRP-FAS* primers in Langi, Opus, Quest, Reiqiz, *O. australiensis* and *O. meridionalis* suggested that the hybridising sequences were imperfect matches to the primers in these varieties/species.

### Rice serpin gene expression data from published microarray and proteomics studies

The Rice GE: Gene Expression Analysis microarray data from the Salk Institute Genomic Analysis Laboratory (SIGnAL) was examined for 12 of the 14 full-length serpin genes [22]; genes *OsSRP-GRA* (Os11g12420) and *-GMS* (Os11g12520) were not represented among the genes in the microarray. Only some of the serpin genes displayed levels of expression above background noise (i.e. values >500). The highest expression levels for all serpin genes in the microarray data were found for *OsSRP-LGC* (Os01g56010) in seeds during late development; i.e. Stage 4 (11–20 dap) and Stage 5 (21–29 dap), mirroring the expression of the barley-grain serpins BSZ4 (HorvuZ4) and BSZ7 (HorvuZ7) [49,50]. Significant expression of this gene was also observed for rice roots treated with transzeatin (a cytokinin) after 30 min and still after 120 min, hinting at involvement of serpins in processes relating to cell division, consistent with the results obtained for *AtSRP2* (At2g14540) and *AtSRP3* (At1g64030) in *Arabidopsis*. *OsSPR-LRS* (Os04g41419) was expressed at substantial levels (values >500) for the majority of the conditions included in the SIGnAL microarray, with expression associated with grain development increasing from Stage 1 (0–2 dap) through to Stage 3 (5–10 dap) and then falling away through Stages 4 and 5. Thus it appears *OsSRP-LRS* was expressed at an earlier stage of grain development than was the *OsSRP-LGC* gene. *OsSRP-PLP* (Os11g11500) was associated with significant levels of expression in the shoot apical meristem (SAM) and at only the latest stage in grain development (Stage 5). It was also expressed at substantial levels (values >500) in the ovule. *OsSRP-LLS* (Os11g13530) was expressed at low levels (values <500) except that expression was higher in Stage 4 of grain development (although at a value associated with a large estimate of error).

*OsSRP-QKG* (Os01g16200), *-FRS* (Os03g41438), *-PTY* (Os04g45110), *-PGY* (Os04g45120), *-PSG* (Os11g11760) and *-FAS* (Os11g12460) were associated with expression values below background noise for all conditions and thus no confident conclusions could be drawn regarding differential expression for these genes.

Proteomics experiments have identified serpins OsSRP-LGC in root and OsSRP-LRS in seed using MudPIT analysis [51]. Overall there is rather little proteomics evidence for the expression of rice serpin genes. This is somewhat surprising considering the high levels of expression of some of the rice serpin genes at the transcript level, particularly *OsSRP-LRS*. It is possible that some rice serpin transcripts (while abundant) are poorly translated, as suggested for the LR serpin gene in barley (encoding BSZx) [52].

### Basal expression analysis of eight rice serpin genes in callus, developing seedlings, mature tissues and developing seeds

Semi-quantitative RT-PCR experiments were performed as a prelude to real-time (qRT-PCR) experiments. These two forms of transcript analysis were run with independent samples (plants grown independently).

**Figure 5:**
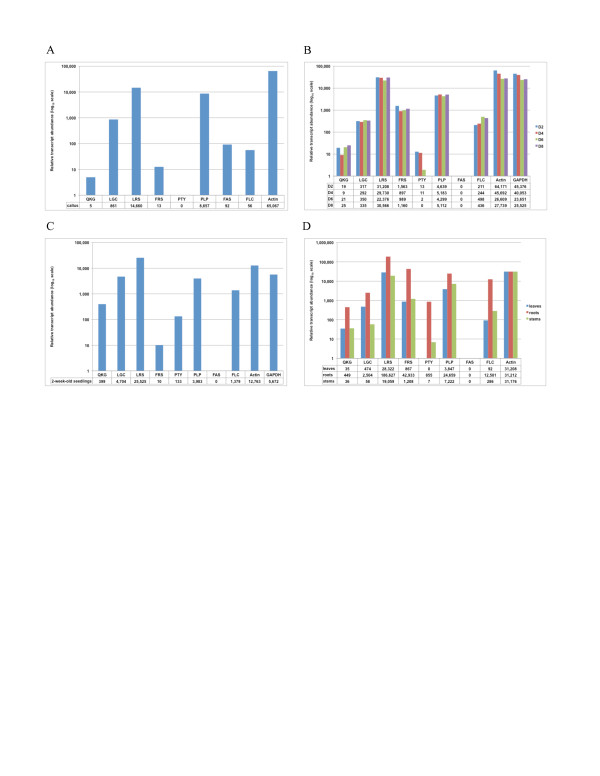
**Basal expression of rice serpin genes.** Real-time RT-PCR was performed using a LightCycler®. Relative transcript abundance is based on CP 20 = 100,000 units. *Actin* was used as a control. **A.** Expression in callus tissue. Total RNA was isolated from callus grown on callus-induction medium. **B.** Expression in developing seedlings. Total RNA was isolated from whole seedlings grown on solid growth medium at 2, 4, 6 and 8 d after germination. **C.** Expression in 2-week-old seedlings. Total RNA was isolated from whole seedlings grown on solid growth medium. **D.** Expression in organs of mature plants. Total RNA was isolated from leaves, roots and stems of mature plants grown in soil.

Real-time analysis of serpin gene expression in callus tissue showed that *OsSRP-LRS* and *-PLP* were the most highly expressed, followed by *OsSRP-LGC* (Figure 5A). *OsSRP-FAS*, -*FLC*, *-FRS* and *-QKG* were scarcely expressed in callus tissue and no expression was detected for *OsSRP-PTY* (CP >36). The pattern of serpin gene expression in callus tissue was very similar to that of 2-d-old developing seedlings (Figure 5B), for which transcript levels relative to *Actin* were almost the same as in callus.

For whole seedlings during development, semi-quantitative RT-PCR using 35 cycles showed that serpin genes *OsSRP-QKG*, *-LGC*, *-LRS*, *-FRS*, *-PTY*, *-PLP* and *-FLC* were expressed in seedlings at all stages examined (2, 4, 6 and 8 d after germination; results not shown). *OsSRP-LRS* and *-PLP* gave product-band intensities higher than those for any of the other serpin genes and similar to those for *Actin*. *OsSRP-QKG*, *-LGC*, *-FRS*, *-PTY* and *-FLC* appeared to be expressed at low levels during seedling development and *OsSRP-FAS* did not appear to be expressed (results not shown). The semi-quantitative data concurred with results of the real-time analysis (Figure 5B). With the exceptions of *OsSRP-PTY* and *-FLC*, the expression patterns of the eight selected rice serpin genes did not change significantly during the first 8 d of seedling development. The very low level of *OsSRP-PTY* expression decreased almost six-fold between D4 and D6 and was not detected at D8, although these levels of expression were near the limits of detection. In contrast, *OsSRP-FLC* transcript was slightly more abundant in D6 and D8 than it was in D2 and D4. No expression (CP >36) was detected for *OsSRP-FAS* in the course of early seedling development (Figure 5B).

Real-time analysis of basal expression of the eight serpins in 2-week-old seedlings showed that, as in younger seedlings (Figure 5B), *OsSRP-LRS* was the most abundantly expressed rice serpin gene (Figure 5C). In 2-week-old seedlings the expression level of *OsSRP-LRS* was even higher than those of the housekeeping genes, *Actin* and *GAPDH*. Other relatively highly expressed rice serpin genes in 2-week-old rice seedlings were *OsSRP-LGC* and *-PLP*. *OsSRP-LGC* expression was an order-of-magnitude higher in 2-week-old seedlings than it was in 2 to 8-d-old seedlings, whereas *OsSRP-FRS* expression was an order of magnitude lower in 2-week-old seedlings (Figure 5C) than it was in 2 to 8-d-old developing seedlings (Figure 5B).

For roots, root tips, stems and leaves of mature rice plants, semi-quantitative RT-PCR using 35 cycles indicated that *OsSRP-LGC*, *-LRS* and *-PLP* were expressed at higher levels than any of the other serpin genes (results not shown). *OsSRP-LRS* gave product-band intensities comparable with those of *Actin*, while *OsSRP-QKG* appeared to be expressed in all of the mature tissues but at a very low level (extremely faint bands). *OsSRP-FRS* appeared to be expressed at a higher level in the stem than in the other tissues and *OsSRP-FLC* was more highly expressed in the root tip, while *OsSRP-PTY* appeared to be expressed only in the root tips. *OsSRP-FAS* did not appear to be expressed in any of the mature tissues (results not shown). The semi-quantitative data were supported by results of the real-time analysis (Figure 5D). *OsSRP-LRS* was the most abundantly expressed rice serpin gene in all three organs, with *OsSRP-FRS* and *-PLP* being the two other highly expressed serpin genes. *OsSRP-FLC*, *-LGC*, *-QKG* and *-PTY* were expressed at very low levels. All serpin genes were expressed at their highest levels in roots when expression levels were normalised to those of *Actin*. No expression signal was detected for *OsSRP-PTY* (CP: >36) in leaves and (again) for *OsSRP-FAS* in any of the organs (Figure 5D).

**Figure 6:**
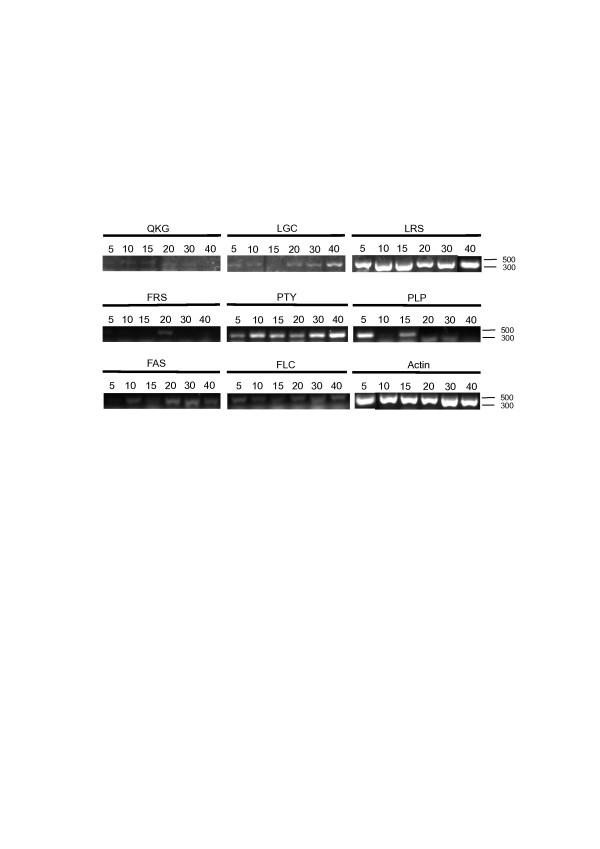
**Expression of eight serpin genes in developing seeds.** Semi-quantitative RT-PCR was used to study the expression of eight serpin genes (labelled according to P2–P1^′^ sequence) in *O. sativa* cv. Nipponbare developing seeds collected 5, 10, 15, 20, 30 and 40 d after anthesis. *Actin* was used as a control.

Serpin gene expression was analysed using semi-quantitative RT-PCR in developing seeds at 5, 10, 15, 20, 30 and 40 d post anthesis (Figure 6). *OsSRP-LRS* was expressed during the entire seed development process at about the same level as *Actin*. *OsSRP-PTY* was also strongly expressed during seed development. *OsSRP-PLP* appeared to be expressed at a relatively high level 5 d after anthesis and then at a lower level as the seed matured. There was a low level of expression of *OsSRP-QKG*, *-LGC*, *-FRS*, *-FAS* and *-FLC* in most stages of seed development (Figure 6).

## Discussion

### Plant serpin nomenclature

The assignment of reactive-centre residues in the new alternative names for the rice serpins is based on canonical positions, which means the P1 residue is identified by counting 17 residues from the highly conserved Glu (normally at P17). These assignments are tentative because some animal serpins have 16 residues between the conserved Glu and the experimentally determined P1 residue. For the vast majority of serpins, however, the physiological target proteinase is expected to cleave at the canonical P1, not at P2 (or elsewhere). We recommend that the terminology of a serpin (using the suggested nomenclature) would be changed if the physiological target proteinase were found to cleave at a residue other than the canonical P1. We also recommend that the terminology would *not* change on the basis of results from testing non-physiological proteases against the serpins (e.g. a mammalian chymotrypsin *versus* a rice serpin). In summary, the name of the serpin would ultimately depend on experimental evidence for the identity of the cleavage site but only with a proteinase that was shown to be the major physiological target. Any change in the suggested nomenclature remains a long-term prospect because currently there is only a single serpin-proteinase partnership established *in vivo* in plants (Lampl et al., 2010).

The reactive centre P2-P1′ residues are different in each of the rice serpins (Additional file 1: Table S1). The P1 residues include positively charged (Arg and Lys), small (Ser and Ala) and medium-sized hydrophobic residues (Met and Leu). For the putatively inhibitory serpins, the P2 residues are Gln, Leu, Phe, Pro and Gly—none being charged. All of the inhibitory serpins have a small residue (Ala, Cys, Gly, Ser) at P1′ (Additional file 1: Table S1), consistent with the majority of animal serpins.

The naming system adopted in this paper could be extended to other plant species to enable easy matching or differentiation between serpins with identical or similar reactive-centre sequences. If a species contained more than one serpin with the same P2-P1′ sequence, the serpins could be named in the same way but with a number after the P2-P1′ designation. Meanwhile, there is a danger that confusion may arise if researchers name the first serpin to be characterised in any particular plant species using a two-letter abbreviation for the species and “Serpin1”; e.g. PsSerpin1 for pea (*Pisum sativum*) without reference to similarity to AtSerpin1.

Until homologous serpins in a range of plant species are shown to have the same function, it is unlikely the systematic naming system based on the (exclusive) membership of plant serpins to “Clade P” (among serpins generally), as proposed earlier [48], will be adopted. When that time arrives, functionally characterised, conserved plant serpins might be named SERPINP1, SERPINP2, etc., in the same fashion as (for example) the animal serpin α_1_-antitrypsin, which belongs to “Clade A”, is named SERPINA1.

### *Phylogeny of the rice serpins*

With the exception of the trichotomy at the base, the phylogenetic analysis produced a tree (Figure 3) suggesting varying levels of relatedness among the 14 rice serpin genes. Fine branching on the right of the phylogenetic tree with bootstrap values of 848 and 1000 showed closely related serpins that presumably resulted from relatively recent gene duplications. The differences in the reactive-centre sequences of these otherwise closely related serpins might reflect a need to broaden the inhibitory specificity of the rice serpin complement for defence against digestive proteinases from insects or pathogens.

### Conservation of serpin genes in Oryza

Genomic PCR using Nipponbare primers suggested that many of the eight serpin genes tested are similar in the rices examined to those in Nipponbare (Figure 4). The absence of a product for a particular gene does not imply that the particular rice does not contain this gene; rather, it indicates that if the gene is present it has changed substantially so that primers are unable to anneal to it. It is noteworthy, however, that *Oryza australiensis* gave PCR amplicons for the fewest serpin primers, consistent with its EE genome (cf. AA genome in *O*. *sativa* and *O*. *meridionalis*).

It is likely that some plant serpins are involved in the regulation of endogenous proteinases while others act directly to inhibit digestive proteinases of insects or pathogens [21,22]. We would expect the reactive centres of the former serpins to be more highly conserved than those of the latter because the pests and pathogens that attack plants in distinct environments would presumably employ different digestive proteinases with distinct proteolytic specificities.

### Comparison of the Arabidopsis and rice serpin complements

Genomic comparisons between *Arabidopsis thaliana* and rice are often instructive, as these model species represent the eudicots and monocots, respectively, and therefore almost all flowering plants. The *Arabidopsis* genome has eight genes encoding full-length serpins, compared to the 14 in the rice (*Oryza sativa* cv. Nipponbare) genome. We compared the diversity of reactive centres between these two species to determine the degree of identity of the putative inhibitory specificity of the serpins present (Table 3). For the putatively inhibitory serpins of both rice (11) and *Arabidopsis* (8), the predicted P1 residues have a range of physico-chemical properties but the serpin reactive centres of the two species do not match to a great extent (Table 3). For example, while four serpins in both rice and *Arabidopsis* have small residues at P1, *Arabidopsis* has one serpin with a negatively charged P1 residue (Asp), whereas rice has none. This serpin might conceivably inhibit a protease with caspase-like activity, such as the recently characterised subtilisin-like enzyme, phytaspase [53]. Unlike rice, *Arabidopsis* has one serpin with a Gln at P1, a polar residue found in most of the grain serpins in wheat [34] and rye [54] and also in specific serpins from several other plant species such as cotton [55]. These differences in serpin complement may make it difficult to predict the function of a particular serpin in rice based on information obtained from *Arabidopsis* or *vice versa*. The clear exception to this is that both *Arabidopsis* and rice contain a single “LR” serpin, namely AtSerpin1 and OsSRP-LRS, respectively, and rice also contains the serpin OsSRP-FRS with a very similar reactive centre. Two other rice serpins, OsSRP-QKG and –GRA, have positively charged residues at P1 but have no matches in the *Arabidopsis* genome (Table 3).

**Table 3:** **Comparison of *Arabidopsis *and rice serpin reactive centres**

**P1 residue type**	**Rice locus (P2-P1′)**	***Arabidopsis*locus (P2-P1′)**
Small (A, C, G, S, T)	Os01g56010 (L**G**C)	At1g64030 (G**C**S) (AtSRP3)
	Os11g11760 (P**S**G)	At2g14540 (T**G**S) (AtSRP2)
	Os11g12410 (G**A**A)	At2g25240 (C**T**S)
	Os11g12460 (F**A**S)	At2g35580 (G**C**R)
Medium & large hydrophobic (F, I, L, M, P, V, W, Y)	Os11g12520 (G**M**S)	At1g62170 (Y**L**G)
	Os11g13530 (L**L**S)	
	Os11g13540 (F**L**C)	
Polar (H, N, Q)		At2g26390 (P**Q**C)
Negatively charged (D, E)		At3g45220 (K**D**M)
Positively charged (K, R)	Os01g16200 (Q**K**G)	At1g47710 (L**R**G) (AtSerpin1)
	Os03g41419 (L**R**S)	
	Os03g41438 (F**R**S)	
	Os11g12420 (G**R**A)	

Amino-acid residues are divided into groups based on physico-chemical properties [47]. The P1 residue is shown in bold. Putatively non-inhibitory serpins OsSRP-PLP, -PTY and –PGY [22] are excluded from the table.

Notwithstanding the somewhat greater reactive-centre diversity of the *Arabidopsis* serpins compared to that of rice, the substantial differences in the reactive centres of the rice serpins are reminiscent of the oat-grain serpins [32] and unlike the glutamine-rich reactive centres in serpins of wheat [34] and rye [54] grain.

### Expression of rice serpin genes during development

*OsSRP-LRS*, *-PLP*, *-FRS* and *-LGC* were expressed at much higher levels than those of the other serpin genes (*OsSRP-FAS*, *-FLC, -PTY* and *-QKG)* at different developmental stages and tissues (Figure 5A–D). With the exception of *OsSRP-FRS*, the identity of the highly expressed genes matched closely to those with greatest expression levels reported in the Rice GE: Gene Expression Analysis microarray data. A great range of basal expression levels was also found among six *Arabidopsis* serpin genes in a previous study [39]. A substantial range of expression levels has also seen among serpins detected at the protein level in mature cereal grains [32,34,54]. Thus different serpin genes within a single plant species feature promoters of vastly different strengths for basal expression.

Since the serpin genes are expressed in callus (Figure 5A), which is undifferentiated tissue, the serpins themselves are unlikely to be required only for processes involving cell differentiation. Moreover, since the pattern of expression among the eight serpin genes is similar in callus to that in plant tissues (i.e. in differentiated cells) it suggests that none of the serpins is required at substantially different levels for any processes found only in differentiated cells under basal conditions.

All selected serpin genes except *OsSRP-FAS* (no expression signal detected) were expressed at highest levels (relative to *Actin*) in roots (Figure 5D), which might be due to the involvement of one or more of the serpins in direct defence against pest/pathogen (exogenous) proteinases from soil-borne organisms. It is conceivable that higher concentrations are needed to protect against soil-borne pests/pathogens than more dispersed pathogens that attack shoot tissues. Additionally, if the serpins (or at least a subset of them) are present to regulate endogenous proteases, perhaps the specific proteinases they regulate are in greater abundance in the roots than in the shoots.

The range of expression levels observed for the rice serpin genes might be due to a need for some serpins to play a role as defensive shields rather than (or as well as) acting as regulatory proteins, and hence to be present at relatively high concentrations [21,22]. As raised earlier, transcription of some of the serpin genes might produce mRNA molecules that are relatively poorly translated. Another reason may be that some of the serpin genes that are expressed weakly under basal conditions are substantially up-regulated by specific stresses or stages of plant development. Finally, some of the serpins might be required at relatively high concentrations under basal conditions (for example, to inhibit a specific endogenous proteinase) but at much lower levels when the target proteinase is required in the cell. Presumably each serpin has a defined half-life in the cell and thus if transcription is lowered, the concentration of serpin eventually falls.

### OsSRP-LRS and the possible functions of LR serpins in plants

The P2-P1^′^ sequence of OsSRP-LRS is an example of the most highly conserved reactive centre among serpins in the Plant Kingdom [22]. P2-P1′ Leu-Arg-Xaa (where Xaa is a small residue) is present in at least one serpin in a large range of plant species (including AtSerpin1 from *Arabidopsis*) and perhaps in all plants [22].

*OsSRP-LRS* was expressed at a high level of mRNA at several developmental stages and in a range of tissues (consistent with the Rice GE: Gene Expression Analysis microarray data), possibly due to an as yet undefined constitutive function of the gene product. It may be that OsSRP-LRS is normally required to inhibit a protease involved in a specific stress response (when the stress is absent), as found for the *Drosophila* serpin, Spn43Ac, which negatively regulates a Toll signalling pathway controlling production of an anti-fungal peptide [56]. Another possibility is that *OsSRP-LRS* mRNA might be translated only under specific stress conditions, allowing the protein to be produced quickly from the abundant transcript.

*OsSRP-LRS* may function by inhibiting a digestive proteinase(s) in insects. Since the LR serpin from barley, BSZx, is known to inhibit proteases of different specificities at overlapping reactive centres *in vitro*[30], there is the strong possibility that OsSRP-LRS could also inhibit proteinases (including exogenous enzymes from pests) of different specificity; i.e. proteases with trypsin-like specificity at P1 Arg and chymotrypsin-like specificity at the canonical P2 Leu. *OsSRP-LRS* is likely to target the cysteine proteinase, oryzain, *in vivo*, as this enzyme is a putative orthologue of *Arabidopsis* RD21, the major proteinase target of AtSerpin1 [7]. The identities of the target proteinases for the other rice serpins remain unknown.

OsSRP-LRS has been shown to be present at the base of tillers in higher abundance in a relatively high-tillering rice cultivar than in a cultivar that produces a relatively low number of tillers [46]. This suggested that this serpin might be involved in the regulation of tiller development. Unfortunately, this study was performed on only two cultivars—one high-tillering and one low-tillering rice—and thus the conclusions drawn need to be validated with a greater number of cultivars.

### OsSRP-PLP and the possible functions of non-inhibitory serpins in plants

**Figure 7:**
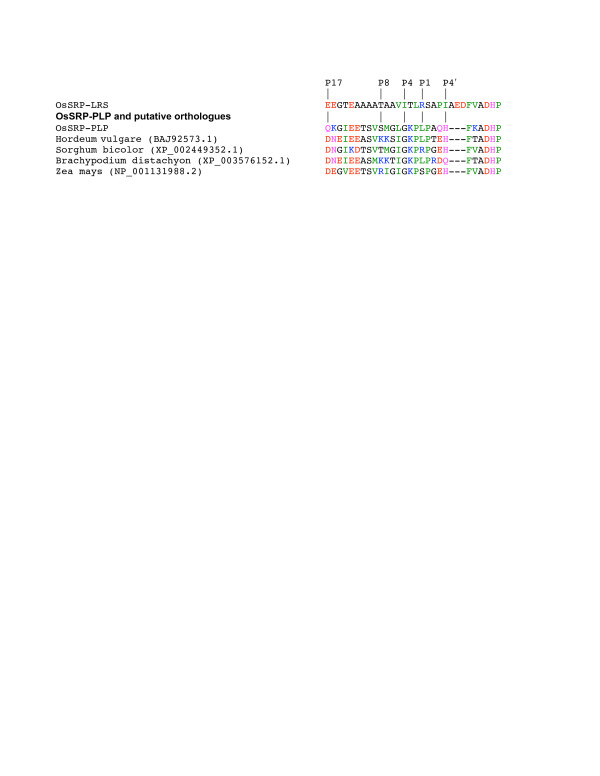
**Amino-acid sequence alignment of the reactive centre loop (RCL) sequences of putative orthologues of OsSRP-PLP in several grass species.** BLAST-P searches of the non-redundant database at NCBI were conducted with the full-length OsSRP-PLP as the query sequence (09 Dec 2011). Accession numbers for the non-rice serpins are given in brackets after the species name. The RCL sequence of OsSRP-LRS is included for comparison. Refer to Figure 1 for residue colour code.

*OsSRP-PLP* was found to be the second most highly expressed serpin gene in developing seedlings, leaves and stems and callus (after *OsSRP-LRS*). These results, combined with the ubiquitous expression of *OsSRP-PLP* based on ESTs, strongly suggest that non-inhibitory serpins have functions in plants, of which there are many possibilities. Non-inhibitory serpins in animals act in diverse roles such as hormone carriage, as performed by corticosteroid binding globulin (CBG or SERPINA6)) and thyroxine binding globulin (TBG or SERPINA7), tumour suppression (maspin or SERPINB5), chaperone activity (HSP47 or SERPINH1) and protein storage (ovalbumin) [57]. The hinge region and reactive centre in the RCL of OsSRP-PLP are surprisingly well conserved in putative orthologues in other grass species (Figure 7), as observed earlier [22]. The conservation of the reactive centre (P4–P1′ GKPXP, where X is any residue) suggests that cleavage by a specific proteinase may be part of the mechanism by which this serpin functions.

Only some of the functions of non-inhibitory serpins in animals are possibilities for non-inhibitory serpins in plants. Plants use a substantial range of hormone (growth regulator) molecules, including brassinosteroids [58], which could conceivably bind to a site on a plant serpin. As plant serpins have already been shown to be present in the phloem sap [36] and to be graft-transmissible [59], there is the possibility that a hormone could then be transported in the phloem via binding to a serpin (and then released following RCL cleavage), as occurs for thyroxin in the blood [20]. While plants do not produce collagen (the protein acted on by HSP47), they do produce many extracellular proteins that need to be properly folded; thus chaperone activity is a possibility for the function of non-inhibitory plant serpins. Protein storage is another possibility for the function of OsSRP-PLP, which is the most likely function for the egg-white serpin, ovalbumin.

## Conclusions

Models for the 14 genes encoding full-length serpins in the *Oryza sativa* cv. Nipponbare genome were confirmed or revised. Each of the 11 putatively inhibitory serpins has a unique reactive centre P2-P1′ sequence although, of these, four have positively charged residues (Arg or Lys) at P1. Based on sequence analysis, one of the other three serpins, OsSRP-PLP, is very likely a non-inhibitory serpin while the non-inhibitory nature of the other two serpins (OsSRP-PTY and -PGY) is less certain. An amino-acid alignment (Figure 1) was used to construct a neighbour-joining phylogenetic tree (Figure 3), which indicated that 10 of the 14 serpins belong to a single clade. It also strongly suggested some of the serpin genes have arisen through relatively recent gene duplications.

At least one example of an LR serpin appears to be expressed in all plant species examined [22], including rice and *Arabidopsis*, and both these species have four serpins with small residues (Ala, Cys, Gly, Ser, Thr) at P1. Otherwise, however, the complement of serpins in rice is quite different to that found in *Arabidopsis* (Table 3). While rice has a greater number of inhibitory serpins, the greater diversity of the *Arabidopsis* P1 residues suggests specific *Arabidopsis* serpins may target proteinases that are not targeted in rice.

Amongst the serpin genes investigated, *OsSRP-LRS* and *-PLP* were found to be expressed at highest levels in callus tissue (Figure 5A), during early stages of seedling development (Figure 5B), in older seedlings (Figure 5C) and in organs of mature rice plants (Figure 5D). In the latter, *OsSRP-LRS* was expressed at highest levels amongst the serpin genes investigated. All selected serpin genes except *OsSRP-FAS* (no expression signal detected) were expressed at highest levels relative to *Actin* in roots. *OsSRP-LRS* appears to behave like a housekeeping gene in that it is constitutively expressed under basal conditions but may be involved in regulation of oryzain activity in stress responses in a manner equivalent to the interaction between AtSerpin1 and RD21 in *Arabidopsis*. There is enormous scope for future studies to provide further functional information for the rice serpins.

## Abbreviations

aa: Amino-acid residues; CP value: Crossing-point value (identical to Ct (crossing threshold) value); MS media: Murashige & Skoog media; MudPIT: Multidimensional Protein Identification Technology; RCL: Reactive-Centre Loop.

## Competing interests

The authors declare no competing interests.

## Authors’ contributions

BJA directed all aspects of growing the rice plants and assisted with data analysis. RAE designed and conducted the real-time RT-PCR experiments and replicates of the semi-quantitative RT-PCR experiments. THR and SEF designed the semi-quantitative RT-PCR and genomic PCR experiments and SEF conducted them. J-WA assisted in the design of all molecular biology aspects of the project. THR conducted the sequence and phylogenetic analyses with assistance from SEF. THR conceived the research, designed the new serpin nomenclature, assessed the data and wrote the manuscript with assistance from the other authors. All authors read and approved the final manuscript.

## Supplementary Material

Additional file 1 **Table S1.** Serpins in rice (*Oryza sativa* cv. Nipponbare). Serpin loci from the Rice Genome Annotation Project (http://rice.plantbiology.msu.edu/) were matched with those from NCBI (which uses a different loci system). The identification of loci in NCBI was performed by comparing the protein sequences in the Rice Genome Annotation Project database to protein sequences in the NCBI database using the BLASTP program. Other loci were identified using the UniProtKB database (http://www.uniprot.org/help/uniprotkb) by searching using the word “serpin” and then checking the identity of the hits based on amino-acid sequence. Click here for file
