# Supplementary material for: Serpins in rice: protein sequence analysis, phylogeny and gene expression during development
Source: BMC Genomics. 2012 Sep 4;13:449. doi: 10.1186/1471-2164-13-449 (PMC3534287; doi:10.1186/1471-2164-13-449)
Supplement: Additional file 1 — Table S1. Serpins in rice (Oryza sativa cv. Nipponbare). Serpin loci from the Rice Genome Annotation Project (http://rice.plantbiology.msu.edu/) were matched with those from NCBI (which uses a different loci system). The identification of loci in NCBI was performed by comparing the protein sequences in the Rice Genome Annotation Project database to protein sequences in the NCBI database using the BLASTP program. Other loci were identified using the UniProtKB database (http://www.uniprot.org/help/uniprotkb) by searching using the word “serpin” and then checking the identity of the hits based on amino-acid sequence. [file 1471-2164-13-449-S1.doc]

Additional file 1: Table S1: Serpins in rice (*Oryza sativa* cv. Nipponbare). Serpin loci from the Rice Genome Annotation Project (http://rice.plantbiology.msu.edu/) were matched with those from NCBI (which uses a different loci system). The identification of loci in NCBI was performed by comparing the protein sequences in the Rice Genome Annotation Project database to protein sequences in the NCBI database using the BLASTP program. Other loci were identified using the UniProtKB database (http://www.uniprot.org/help/uniprotkb) by searching using the word “serpin” and then checking the identity of the hits based on amino-acid sequence.

| **MSU locus**  **number** | **NCBI locus**  **number** | **NCBI protein**  **accession number** | **Alternative serpin name** | **New serpin name** | **RCL sequence**  **(P2-P1′ residues in bold)**  **P14 P8 P1 P4′**  | | || | **Protein length (aa)** |
| --- | --- | --- | --- | --- | --- | --- |
| Os01g16200 | Os01g0267300 | Q5NBM0 | OrysaZ12 | OsSRP-QKG | ELGTVAAASTAVVMM**QKG**SSL------PPVDF  | | || | 398 |
| Os01g56010 | Os01g0765400 | Q94DW6 | OrysaZ1 | OsSRP-LGC | EEGTEAAAATAVVMT**LGC**AAP--SAPVHVVDF  | | || | 411 |
| Os03g41419 | Os03g0610650 | Q75H81 | OrysaZxa | OsSRP-LRS  (OsSerpin1) | EEGTEAAAATAAVIT**LRS**API-------AEDF  | | || | 396 |
| Os03g41438 | Os03g0610800 | Q10GX0 | OrysaZxb | OsSRP-FRS | EEGTEAAAASAAVVS**FRS**APV-------TVDF  | | || | 405 |
| Os04g45110 | Os04g0533700 | Q7XMK1 | OrysaZ10 | OsSRP-PTY | EEGTTAVEATYSCCSPTYSGP-ESPKPRPMSF  | | || | 392 |
| Os04g45120 | Os04g0533700 | Q7XMK0 | OrysaZ11 | OsSRP-PGY | EEGTTAVEAMYSPSSPGYSPGYQPPRPPPMSF  | | || | 385 |
| Os11g11500 | Os11g0222200 | Q2R8P2 | OrysaZ9 | OsSRP-PLP | QKGIEETSVSMGLGKPLPAQH----------F  | | || | 439 |
| Os11g11760 | Os11g0224800 | Q53P09 | OrysaZ8 | OsSRP-PSG | EEGTVAAAATMTRML**PSG**VPPPPVDFVAEHPF  | | || | 417 |
| Os11g12410 | Os11g0230400 | Q53Q31 | OrysaZ6a | OsSRP-GAA | EEGTEAAAATAVLME**GAA**RYA--PPPPPREDF  | | || | 393 |
| Os11g12420 | Os11g0230700 | Q53Q32 | OrysaZ6b | OsSRP-GRA | EEGTEVAAATVVIMK**GRA**RRP--SPAPAPVDF  | | || | 398 |
| Os11g12460 | Os11g0231200 | Q53MD1 | OrysaZ6c | OsSRP-FAS | EEGTEAAAATAVCLT**FAS**AAP-SSRRPARVDF  | | || | 415 |
| Os11g12520 | Os11g0232000 | Q53MD3 | OrysaZ5 | OsSRP-GMS | EEGTEAAASAINMVC**GMS**MTP--EPRPVPVDF  | | || | 445 |
| Os11g13530 | Os11g0239000 | Q53KS8 | OrysaZ2a | OsSRP-LLS | EEGTEAAASTACTIR**LLS**MSY-------PEDF  | | || | 424 |
| Os11g13540 | Os11g0239200 | Q53KS9 | OrysaZ2b | OsSRP-FLC | EEGTEAAAATACTMK**FLC**LTL-----TSPVDF  | | || | 404 |
